# Temperature control after cardiac arrest

**DOI:** 10.1186/s13054-022-04238-z

**Published:** 2022-11-24

**Authors:** Claudio Sandroni, Daniele Natalini, Jerry P. Nolan

**Affiliations:** 1 Università Cattolica del Sacro Cuore, Largo Francesco Vito 1, 00168 Rome, Italy; 2 Department of Intensive Care, Emergency Medicine, and Anesthesiology, Fondazione Policlinico Universitario A. Gemelli, IRCCS. Largo A. Gemelli 8, 00168 Rome, Italy; 3 Warwick Clinical Trials Unit, Warwick Medical School, Warwick University, Gibbet Hill, Coventry, CV4 7AL UK; 4 Department of Anaesthesia and Intensive Care Medicine, Royal United Hospital, Bath, BA1 3NG UK; 5 Department of Anesthesiology and Intensive Care Medicine, Catholic University of The Sacred Heart. Fondazione ‘Policlinico Universitario A. Gemelli’ IRCCS. L.go F, Vito 1, 00168 Rome, Italy

**Keywords:** Cardiac arrest, Coma, Hypothermia, Hypoxic-ischemic brain injury, Temperature control

## Abstract

Most of the patients who die after cardiac arrest do so because of hypoxic-ischemic brain injury (HIBI). Experimental evidence shows that temperature control targeted at hypothermia mitigates HIBI. In 2002, one randomized trial and one quasi-randomized trial showed that temperature control targeted at 32–34 °C improved neurological outcome and mortality in patients who are comatose after cardiac arrest. However, following the publication of these trials, other studies have questioned the neuroprotective effects of hypothermia. In 2021, the largest study conducted so far on temperature control (the TTM-2 trial) including 1900 adults comatose after resuscitation showed no effect of temperature control targeted at 33 °C compared with normothermia or fever control. A systematic review of 32 trials published between 2001 and 2021 concluded that temperature control with a target of 32–34 °C compared with fever prevention did not result in an improvement in survival (RR 1.08; 95% CI 0.89–1.30) or favorable functional outcome (RR 1.21; 95% CI 0.91–1.61) at 90–180 days after resuscitation. There was substantial heterogeneity across the trials, and the certainty of the evidence was low. Based on these results, the International Liaison Committee on Resuscitation currently recommends monitoring core temperature and actively preventing fever (37.7 °C) for at least 72 h in patients who are comatose after resuscitation from cardiac arrest. Future studies are needed to identify potential patient subgroups who may benefit from temperature control aimed at hypothermia. There are no trials comparing normothermia or fever control with no temperature control after cardiac arrest.

## Background

Cardiac arrest ranks among the most important causes of mortality worldwide. In the USA, almost 90 individuals per 100,000 population are resuscitated from out-of-hospital cardiac arrest (OHCA) [1]. The incidence of in-hospital cardiac arrest (IHCA) has been estimated to be between 0.6 and 5 events per 1000 patient admissions [2, 3]. More than two-thirds of patients resuscitated from OHCA [4], and about one-quarter of those resuscitated from IHCA [5] die of hypoxic-ischemic brain injury (HIBI) [6]. Temperature control has been the most widely studied among the strategies for reducing the severity of HIBI. The present review summarizes the experimental and clinical evidence, the most recent recommendations, and future research directions regarding the use of temperature control after cardiac arrest.

### Pathophysiology and rationale

The human brain is very susceptible to HIBI—consciousness is lost between 4 and 10 s after the arrest of cerebral blood flow and the electroencephalogram becomes isoelectric 10–30 s after circulatory arrest. Irreversible damage to the neurons starts immediately upon cessation of cerebral perfusion because of the lack of inherent energy stores, and it continues after reperfusion due to the release of excitatory amino acids, intracellular calcium influx and release, generation of free radicals, and triggering of apoptosis [6, 7].

Inducing hypothermia before cardiac arrest protects the brain from HIBI; deep hypothermia (body core temperature < 20 °C) enables up to 30–40 min of full circulatory arrest without neurological injury [8] and it has been used since 1955 in cardiac surgery with circulatory arrest [9]. Accidental hypothermia preceding cardiac arrest also confers neuroprotection. In a systematic review that included 210 cases of cardiac arrest related to accidental hypothermia, 153 (73%) survived to hospital discharge. Favorable neurological outcome was reported in 103/105 (89%) survivors. The average body temperature of these patients was 23.9 ± 2.7 °C. Full neurological recovery has been reported in cardiac arrest from accidental hypothermia with body temperatures as low as 13.7 °C after prolonged resuscitation [10].

The potential neuroprotective effects of hypothermia induced after the return of spontaneous circulation (ROSC) from cardiac arrest have been investigated more recently. A systematic review and meta-analysis of experimental evidence included 45 studies published between 1991 and 2019. In these studies, the target temperature ranged from 32 to 36.2 °C in the treatment group and from 36.5 to 39.3 °C in the control group. Notably, the temperatures in the control group were maintained within the normal range for each species used in the model. Results showed that hypothermia reduced mortality by 67% and improved neurological outcomes. These benefits were greater in large animals compared with small animals. Faster cooling rates, lower target temperatures, and shorter delays to starting cooling were independently associated with an increasing effect size [11].

### Clinical trials on targeted temperature management

Two pilot trials published in 1997 and 2000 showed the feasibility of cooling post-cardiac arrest patients in the emergency department [12, 13]. In 2002, two clinical trials, one randomized [14] and one pseudorandomized [15], documented improved outcomes with mild hypothermia in patients initially comatose after OHCA with an initial shockable rhythm. In their randomized clinical trial [14], the Hypothermia After Cardiac Arrest (HACA) Study Group reported a higher rate of favorable functional outcome (Cerebral Performance Category [CPC] 1–2) at 6 months with mild hypothermia (32 °C–34 °C) for 24 h compared with normothermia (hypothermia 75/136 [55%] versus normothermia 54/137 [39%]; risk ratio [RR] 1.40; 95% confidence interval [95%CI] 1.08 to 1.81) [14]. The smaller trial documented a higher rate of discharge to home or to a rehabilitation facility after treatment with hypothermia (33 °C) for 12 h compared with normothermia (hypothermia 21/ 43 [(49%] versus normothermia 9/34 [26%]; *p* = 0.046)[15]. The ILCOR ALS Task Force considered these studies and the supporting data from animal studies [16] and published an advisory statement in 2003 recommending that unconscious adult OHCA patients with spontaneous circulation should be cooled for 32–34 °C for 12–24 h when the initial rhythm was ventricular fibrillation (VF) [17]. The Task Force also stated that “such cooling may also be beneficial for other rhythms or in-hospital cardiac arrest.” Some individuals have highlighted significant limitations that placed these early trials at high-risk of bias [18]. The treating clinicians could not be blinded and in the absence of a strict prognostication protocol, decisions on withdrawal of life-sustaining treatment (WLST) and its timing could have been influenced by the use of mild hypothermia. Use of mild-induced hypothermia is likely to require a longer period of sedation, which may delay WLST decisions, giving patients a greater opportunity to awaken.

To address the potential limitations of the two 2002 clinical trials, the Targeted Temperature Management (TTM) trial investigators randomized 950 unconscious adults after OHCA from cardiac cause and any initial rhythm to temperature control at 33 °C compared with a target of 36 °C for 24 h, followed by slow rewarming at 0.5 °C/h [19]. There was no difference in all-cause mortality (the primary outcome) or 6-month functional outcome. Following this trial, many clinicians aimed for a target temperature of 36 °C in post-cardiac arrest patients, while others continued to aim for 33 °C. Some clinicians abandoned the use of temperature control completely [20]. Analyses of large ICU registries in Australia and New Zealand [21, 22] and in the United Kingdom [23] documented an increase in the lowest temperature of post-cardiac arrest patents in the first 24 h in ICU since publication of the TTM trial. This was associated with an increase in the frequency of fever in the first 24 h. The lowest temperature in the first 24 h was evaluated because this is collected routinely for sickness severity scoring and case mix adjustment. Although the unadjusted mortality rate was higher in the post-TTM period, analyses that removed all temperature-affected variables showed no difference in the adjusted mortality rate between pre- and post-TTM periods. If any of the components of a risk-adjustment model are affected by a treatment intervention—in this case temperature—it invalidates the risk calculation. In these studies, the changes in risk-adjusted mortality mirrored the changes in temperature. It is possible that the decreasing temperatures before publication of the TTM trial were interpreted by the risk models as increasing risk of death and the increasing temperature after publication of the TTM trial interpreted as a decrease in risk of death.

There are several potential reasons for the conflicting results between the two clinical trials in 2002 and the TTM trial of 2013. Unlike the 2002 studies, the TTM trial investigators used a protocolized approach to prognostication: for patients who remained unconscious, a clinician blinded to the group allocation performed a neurological examination 72 h after the end of the intervention and made a recommendation for continuation or WLST. Temperature was not well controlled or not controlled at all in “normothermia” groups of the 2002 trials, and many of these patients developed fever. Post-cardiac arrest fever is associated with a worse outcome [24–26], but whether this causes the worse outcome is uncertain.

In the TTM-2 study, 1900 adults comatose after resuscitation from all-rhythm OHCA of presumed cardiac or unknown cause were randomized to temperature control at 33 °C or normothermia with early treatment of fever (≥ 37.7 °C) followed by temperature control at 37.5 °C [27]. After the intervention period of 40 h, normothermia was maintained until 72 h after randomization in patients who remained sedated or comatose. The primary outcome, 6-month mortality, was no different between the groups (50% in the hypothermia group and 48% in the normothermia group (RR 1.04; 95% CI 0.94 to 1.14; *p* = 0.37). Functional outcome at six months was also the same—55% in both groups had moderately severe disability or worse (modified Rankin scale score ≥ 4; RR 1.00; 95% CI 0.92 to 1.09). Arrhythmia resulting in hemodynamic compromise was more common in the hypothermia group than in the normothermia group (24% versus 17%, *p* < 0.001). There was no difference in outcome among several prespecified groups, including time to ROSC (greater or less than 25 min) and initial rhythm.

The TTM investigators have undertaken an individual patient data meta-analysis of the TTM-1 and TTM-2 studies [28]. Patients in the 36 °C group from TTM-1 were combined with those in the normothermia group in TTM-2 and were compared with the patients from the 33 °C groups in both TTM studies. The primary outcome, all-cause mortality at 6 months, occurred in 691 of 1398 participants (49.4%) in the 33 °C group and 666 of 1391 participants (47.9%) in the normothermia group (RR 1.03; 95% CI 0.96 to 1.11; *p* = 0.41). There was no difference in outcomes between several prespecified subgroups, including initial rhythm and time to ROSC (greater or less than 25 min).

### Recent systematic reviews

After the publication of the TTM-2 trial, the Advanced Life Support (ALS) Task Force of the International Liaison Committee on Resuscitation (ILCOR) commissioned a new systematic review and meta-analysis of trials on temperature management in adult patients with HIBI after resuscitation from OHCA or IHCA from all rhythms [29]. The questions addressed by the systematic review were: (1) temperature control at 32–34 °C compared with normothermia/ fever prevention; (2) the specific target of temperature control; (3) the timing of temperature control initiation; (4) the method used for temperature control; (5) the duration of temperature control and (6) the rewarming rate from hypothermia. The authors of the systematic review used the GRADE (Grading of Recommendations Assessment, Development, and Evaluation) approach to assess the certainty of evidence [30]. This was categorized as very low, low, moderate, or high based on risk of bias, imprecision, indirectness, inconsistency, and publication bias [9].

The review included 38 studies representing 32 trials published between 2001 and 2021. Of these, nine trials compared temperature control with a target of 32–34 °C with normothermia, and six were included in the meta-analyses. Temperature control with a target of 32–34 °C did not result in an improvement in survival (RR 1.08; 95% CI 0.89 to 1.30) or favorable functional outcome (RR 1.21; 95% CI 0.91 to 1.61) at 90 to 180 days after resuscitation. There was substantial heterogeneity across the trials, and the certainty of the evidence was low. Three trials assessed different hypothermic temperature targets and found no difference in outcomes between 33 and 36 °C [19] or between 32, 33, and 34 °C (low certainty of evidence). Ten trials comparing prehospital cooling with no prehospital cooling showed no difference in survival (RR 1.01, 95% CI 0.92 to 1.11) or favorable functional outcome at discharge (RR 1.00, 95% CI 0.90 to 1.11) (moderate certainty of evidence). Three trials comparing endovascular with surface cooling showed no difference in survival or neurological outcome to discharge [31–33]. No trials on rewarming strategies were found.

In the same year (2021), a systematic review and network meta-analysis investigated the efficacy and safety of maintaining different temperature targets after cardiac arrest [34]. The review was restricted to OHCA patients and assessed ten trials (total 4218 patients), including the CAPITAL-CHILL trial on hypothermia at 31 °C versus 34 °C, which was not included in the previous review [35]. Its results confirmed that hypothermia at 31–32 °C, 33–34 °C, and 35–36 °C did not improve survival with good functional outcome as compared to normothermia at 37–37.8 °C (odds ratio [OR] 1.30, [0.73 − 2.30], 1.34 [0.92–1.94], and 1.44[0.74–2.80], respectively). The review also assessed the incidence of adverse events for different temperature targets. The incidence of arrhythmias was higher among patients treated with hypothermia at 31–32 °C and 33–34 °C compared with normothermia, with higher odds observed for lower hypothermic temperature targets (OR 3.58 [1.77–7.26] and 1.45 [1.08–1.94], respectively). The incidence of bleeding and pneumonia was not significantly different.

### ILCOR and ERC/ESICM recommendations

Based on the results of the ILCOR-commissioned systematic review, the ILCOR ALS Task Force posted online its recommendations (Table 1) [36, 37].

**Table 1:** **ILCOR Recommendations on temperature control after cardiac arrest. From **[36]

We suggest actively preventing fever by targeting a temperature of 37.5 °C or less for patients who remain comatose after ROSC from cardiac arrest (weak recommendation, low certainty of evidence)
Whether subpopulations of cardiac arrest patients may benefit from targeting hypothermia at 32–34 °C remains uncertain
Comatose patients with mild hypothermia after ROSC should not be actively warmed to achieve normothermia (good practice statement)
We recommend against the routine use of prehospital cooling with rapid infusion of large volumes of cold IV fluid immediately after ROSC (strong recommendation, moderate certainty of evidence)
We suggest surface or endovascular temperature control techniques when temperature control is used in comatose patients after ROSC (weak recommendation, low certainty of evidence)
When a cooling device is used, we suggest using a temperature control device that includes a feedback system based on continuous temperature monitoring to maintain the target temperature (good practice statement)
We suggest active prevention of fever for at least 72 h in post-cardiac arrest patients who remain comatose (good practice statement)

The most significant change compared with previous recommendations was that hypothermia was no longer recommended for the treatment of comatose cardiac arrest survivors. Instead, the Task Force suggested actively preventing fever (defined as a temperature > 37.7 °C) for at least 72 h after ROSC. This recommendation was because, although no difference in neurological outcome was found between patients treated with hypothermia and normothermia or fever prevention, fever prevention probably has fewer side effects and requires fewer resources than hypothermia. In the TTM-2 trial, significantly more patients in the hypothermia group had arrhythmia causing hemodynamic compromise than in the normothermia group (24% vs. 17%, *p* < 0.001). Moreover, in that trial, the majority (54%) of patients in the normothermia group did not require cooling with a device compared with 5% in the hypothermia group [27]. In those patients, the temperature target was maintained using pharmacological measures (acetaminophen), uncovering the body, or lowering ambient temperature.

The ALS ILCOR task force suggested using the term “temperature control” rather than the commonly used term “TTM” to avoid confusion with the TTM-2 randomized controlled trials and preferred using the term “prevention of fever” rather than normothermia. To provide additional clarity for interpreting future clinical trials and evidence reviews, the Task Force proposed and updated terminology for temperature control strategies (Table 2).

**Table 2:** Proposed ILCOR terminology for temperature control interventions

Hypothermic Temperature Control	Active temperature control with the target temperature below the normal range
Normothermic Temperature Control	Active temperature control with the target temperature in the normal range
Fever Prevention Temperature Control	Monitoring temperature and actively preventing and treating temperature above the normal range
No Temperature Control	No protocolized active temperature control strategy

In 2022, the European Resuscitation Council (ERC) and the European Society of Intensive Care Medicine (ESICM) published updated guidelines on temperature control after cardiac arrest [37, 38]. Consistent with the ILCOR consensus document, these guidelines recommend continuous monitoring of core temperature and actively preventing fever for at least 72 h in patients who remain comatose after cardiac arrest.

### Points for discussion and knowledge gaps

Despite insufficient evidence to recommend for or against temperature control at 32–36.8 °C after cardiac arrest, most members of the ILCOR ALS Task Force and the ERC-ESICM panel were keen to leave open this option in some patient categories according to local protocols. One reason was that most patients included in the evidence reviews had been in cardiac arrest with an initial shockable cardiac rhythm and a primary cardiac cause [19, 27, 39]. Other categories of patients in whom temperature control with hypothermia could be more effective may exist. However, these populations have not been identified with certainty. In the HYPERION trial, conducted on 584 patients resuscitated from non-shockable cardiac arrest (27% in-hospital), the rates of 90-day survival with favorable functional outcome were significantly higher after 24-h temperature control targeted at 33 °C versus 37 °C (10.2 vs. 5.7%; *p* = 0.04) [40]. Notably, the HYPERION study had a fragility index of 1, meaning that switching only one patient in the treatment group from good to poor neurological outcome at 90 days would convert the statistically significant difference between groups observed in the study to non-significant. Data from the TTM and the TTM-2 trials showed no benefit from controlled temperature targeted at hypothermia in patients with non-shockable rhythm. An ancillary analysis of the HYPERION trial showed larger differences in favor of temperature control targeted at 33 °C in the subpopulation of patients with in-hospital arrest (16.4% vs. 5.8%; *p* = 0.03)[41]. However, mortality did not differ between the two groups. A recent multicenter trial [42] conducted on 249 patients resuscitated from in-hospital cardiac arrest in Germany showed no benefit of temperature control targeted at 32–34 °C on mortality at six months compared with normothermia.

The presence of confounders, such as a different etiology or comorbidities, may limit the reliability of the first recorded rhythm (shockable vs. non-shockable) as a marker of the severity of HIBI. A better approach could be to investigate the effectiveness of temperature control dose—in terms of target temperature and duration—based on prognostic scores such as the cardiac arrest hospital prognosis (CAHP) score [43] or on direct clinical indices of HIBI severity, such as those currently used as prognostic tests in post-cardiac arrest coma [44, 45]. “Prognostic enrichment” has been advocated to focus randomized trials on patients more likely to benefit from the assessed treatment [46].

A possible reason for the lack of consistent benefit from hypothermia after cardiac arrest is that in the studies conducted so far, the target temperature was reached several hours after ROSC and potentially outside the therapeutic window. In the 45 animal studies that showed a consistent benefit from temperature control targeted at hypothermia after cardiac arrest, hypothermia was induced in 1 h or less after ROSC, and the mean cooling rate was 11 °C/h (range 0.8 °C/h to 27 °C/h) [11]. This cooling rate has never been achieved in any clinical study. In one randomized trial [47], prehospital administration of up to 2 L of 4 °C normal saline after ROSC followed by hospital cooling reduced the time to achieving the target temperature of 34 °C by more than one hour (from 4.2[3.8–4.6] to 3.0[2.6–3.4] hours). However, there was no difference between groups in terms of survival or neurological outcome at discharge. Moreover, the intervention was associated with significantly higher rates of rearrest in the field and pulmonary edema on the first chest X-ray. The 2021 ERC-ESICM Guidelines recommend against the routine use of prehospital cooling with rapid infusion of large volumes of cold IV fluid immediately after ROSC (Table 1).

Two clinical studies [48, 49] showed that intranasal cooling during resuscitation from OHCA achieved significantly shorter times to both tympanic and core target temperature compared with no intranasal cooling, and it was not associated with higher rates of adverse events. Neurological outcome was not different between the study groups. An individual patient data meta-analysis based on these two studies showed that intranasal cooling was associated with increased rates of favorable neurological outcome (34.2% vs. 24.0%; RR 1.43 [95% CI 1.01–2.02]) in patients with initial shockable rhythms [50].

The optimal duration of temperature control after cardiac arrest is still a matter of debate*.* The ILCOR review included only one trial [39], which showed no difference in outcomes between temperature control at 32–34 °C for 24 h compared with 48 h after OHCA. The ongoing multicenter ICECAP study (NCT 04,217,551) in the United States (US) is investigating the effects of different durations [from 6 to 72 h] of controlled temperature targeted at 33 °C on neurological outcome at three months. The 2021 recommendations suggest preventing fever for at least 72 h after ROSC, based on the two TTM trials [19, 27] where the temperature was controlled for at least 72 h in patients who remained sedated or comatose.

There is no universally accepted definition of normothermia. In 2017, a large study based on 243,506 measurements made in a cohort of 35,488 non-infectious adult outpatients in a single academic hospital in the USA showed that the 95% range for body temperature was 35.7 to 37.3 °C, and the 99% range was 35.3 to 37.7 °C [51]. The site of temperature measurements was oral in most patients (88%). Patients assessed in the emergency department were excluded from the study. Whether these ranges can be generalized to core temperatures measured in comatose resuscitated patients is uncertain.

A final knowledge gap concerns the neuroprotective role of normothermia. Although hyperthermia is considered to be detrimental after cardiac arrest, there are no trials comparing normothermia or fever prevention with no temperature control in patients who are comatose after cardiac arrest.

## Conclusions

Twenty years after the publication of the first randomized trials on hypothermia after cardiac arrest, the protective role of temperature control for HIBI is still debated. Given the lack of any consistent evidence in favor of hypothermic temperature control, current guidelines recommend continuous monitoring of core temperature and actively preventing fever (> 37.7 °C) in adult patients who are comatose after resuscitation from cardiac arrest, regardless of cardiac arrest location or initial rhythm. However, the overall certainty of evidence regarding temperature control after cardiac arrest is low. Future studies are warranted to identify patient populations for whom hypothermic temperature control could be beneficial and if normothermia or fever prevention has a neuroprotective effect compared with no temperature control.

## Data Availability

Not Applicable.

## Abbreviations

ALS: Advanced life support
CAHP: Cardiac Arrest Hospital Prognosis Score
CPC: Cerebral performance category
HACA: Hypothermia after cardiac arrest
HIBI: Hypoxic-ischemic brain injury
IHCA: In-hospital cardiac arrest
ILCOR: International Liaison Committee on Resuscitation
OHCA: Out-of-hospital cardiac arrest
ROSC: Return of spontaneous circulation
TTM: Targeted temperature management
VF: Ventricular fibrillation
WLST: Withdrawal of life-sustaining treatment
